# Possible Mechanisms of Dark Tea in Cancer Prevention and Management: A Comprehensive Review

**DOI:** 10.3390/nu15183903

**Published:** 2023-09-07

**Authors:** Huilin Deng, Jia Liu, Ying Xiao, Jian-Lin Wu, Rui Jiao

**Affiliations:** 1 Department of Food Science and Engineering, Institute of Science and Technology, Jinan University, 601 Huangpu Road, Guangzhou 510632, China; 15274066147@163.com (H.D.); 13484879941@163.com (J.L.); 2 Faculty of Medicine, Macau University of Science and Technology, Macau, China; yxiao@must.edu.mo; 3 State Key Laboratory of Quality Research in Chinese Medicine, Macau Institute for Applied Research in Medicine and Health, Macau University of Science and Technology, Macau, China; jlwu@must.edu.mo

**Keywords:** dark tea, tea polyphenols, tea polysaccharide, theabrownin, MAPK/JNK, NF-κB, anti-cancer, dietary advice

## Abstract

Tea is one of the most popular drinks in the world. Dark tea is a kind of post-fermented tea with unique sensory characteristics that is produced by the special fermentation of microorganisms. It contains many bioactive substances, such as tea polyphenols, theabrownin, tea polysaccharides, etc., which have been reported to be beneficial to human health. This paper reviewed the latest research on dark tea’s potential in preventing and managing cancer, and the mechanisms mainly involved anti-oxidation, anti-inflammation, inhibiting cancer cell proliferation, inducing cancer cell apoptosis, inhibiting tumor metastasis, and regulating intestinal flora. The purpose of this review is to accumulate evidence on the anti-cancer effects of dark tea, the corresponding mechanisms and limitations of dark tea for cancer prevention and management, the future prospects, and demanding questions about dark tea’s possible contributions as an anti-cancer adjuvant.

## 1. Introduction

Nowadays, the incidence and death rate of cancer are increasing year by year. Cancer is not only the leading cause of death but also the main obstacle to extending human life in various countries [1]. According to recent statistics from the World Health Organization (WHO), there were 19.3 million new cancer cases and 10 million cancer-related deaths worldwide in 2020. The four most common cancers in the world are lung cancer, female breast cancer, bowel cancer, and prostate cancer, which account for four out of ten of all cancers diagnosed worldwide. By 2040, 27.5 million new cases of cancer are expected to occur globally each year [2]. Given this trend, the prevention and management of cancer have urgently become important issues worldwide. The occurrence of cancer is closely related to people’s lifestyle, dietary habits, environmental changes, genetics, and other factors [3,4]. Previous studies have reported a negative correlation between tea consumption and cancer [5,6].

Tea can be divided into green tea, black tea, oolong tea, white tea, yellow tea, and dark tea according to the different degrees of fermentation. Dark tea belongs to post-fermented tea. According to the origins, processing technology, and different fermentation strains, there are several types of dark tea in China, mainly including Yunnan Pu’er tea, Guangxi Liupao tea, Hunan Fubrick tea, Hubei Qingbrick tea, Sichuan Kangbrick tea, and so on. Different dark teas are different in appearance, aroma, and content of active ingredients. For example, Fu brick tea has a higher content of catechin and flavonoid glycosides, while Pu’er tea has a higher content of catechin derivatives, flavonoids, and alkaloids [7]. Studies have confirmed that dark tea has a high potential for anti-cancer activity for human health [8,9,10,11], and it is recommended as a nutritional supplement for the prevention and management of cancer [12,13,14]. Therefore, this review summarized the possible anti-cancer mechanisms of dark tea in the past 10 years through articles on the Web of Science, PubMed, and Google Scholar. The following string terms are used to search for articles: “dark tea” and “anti-inflammation” or “anti-oxidation” or “anti-cancer”. We explore the potential of dark tea for preventing and managing cancer and also discuss the corresponding mechanisms and limitations.

## 2. Bioactive Components in Dark Tea

Dark tea contains a variety of bioactive compounds, including catechins, phenolic acids, flavonols, flavones, alkaloids, terpenoids, and their derivatives [9]. It has unique flavor substances and active ingredients that are different from black tea, green tea, and oolong tea. Microbial fermentation is believed to be a key factor responsible for shaping the sensory properties and beneficial health effects of dark tea [15].

The polyphenols in dark tea include catechins, flavonoids, anthocyanins, and other compounds, which have biological activities such as anti-oxidation, antisepsis, and anti-inflammation [16,17,18]. Dark tea is also rich in amino acids, especially theanine and γ-aminobutyric acid, which have biological activities such as enhancing immunity, anti-fatigue, improving memory, and calming [19,20,21]. In addition, dark tea contains short- and medium-chain saturated fatty acids, phenolic acids, organic acids, alkaloids, nucleotides, etc. Alkaloids such as caffeine have been reported to lower blood cholesterol, excite nerves, and benefit cardiovascular health [22]. Polysaccharides are also abundant in dark tea and have biological activities such as regulating immunity, lowering blood sugar, and improving intestinal flora. Last but not least, theabrownin, a heterogeneous polyphenolic compound, is one of the most active and abundant pigments in dark tea, and it is also the characteristic constituent of dark tea. In recent ten years, theabrownin in dark tea has been reported to possess various kinds of activities, including anti-oxidation, anti-inflammation, reducing plasma lipids and body weight, anti-cancer, and prevention of diabetes [23,24,25,26].

## 3. Anti-Cancer Effects of Dark Tea and Its Components in Different Cancer Models

Active components in dark tea have been shown to possess anti-cancer effects in different cancer models, such as lung, liver, skin, breast, and pancreatic cancers, in vivo and in vitro, but few population experiments have been conducted.

The water-soluble polysaccharides isolated from the dark brick tea significantly inhibited the proliferation of lung cancer cells A549 and liver cancer cells SMMC7721 [27]. Theabrownin was reported to inhibit the proliferation of lung cancer cells in vitro and in vivo [28]. A new epigallocatechin gallate derivative isolated from Anhua dark tea enhanced the apoptosis of lung cancer cells HCC827-Gef [29]. The extract of Ya’an Tibetan tea has an anti-proliferation effect on the human hepatoma cell line HepG2 [30]. Theabrownin significantly promoted the apoptosis of human melanoma cells A375 and inhibited the growth of zebrafish A375 xenograft tumors [31], and also inhibited the proliferation of Huh7 liver cancer cells by activating the Jun N-terminal Kinase pathway, as well as inhibited the tumor growth in Huh7 xenografted zebrafish [32]. Dark tea contains caffeine, theophylline, and theobromine, and theophylline inhibits the proliferation of breast cancer cells MDA-MB-231 and MCF-7 and cervical cancer cells HeLa by down-regulating the expression of SRSF3 [33]. Pu’er tea extract can inhibit the proliferation of human tongue cancer TCA8113 cells and has been proven to prevent oral mucosa cancer in mice [34]. Dark tea extract inhibited the proliferation of human pancreatic cancer cells SW1990, PANC-1, and human colon adenocarcinoma cells SW1116. After subcutaneous injection of PANC-1 cells into athymic nude mice, dark tea inhibited the growth of cancer cells in a xenograft tumor model [35].

## 4. The Possible Anti-Cancer Mechanisms of Dark Tea

**Figure 1:**
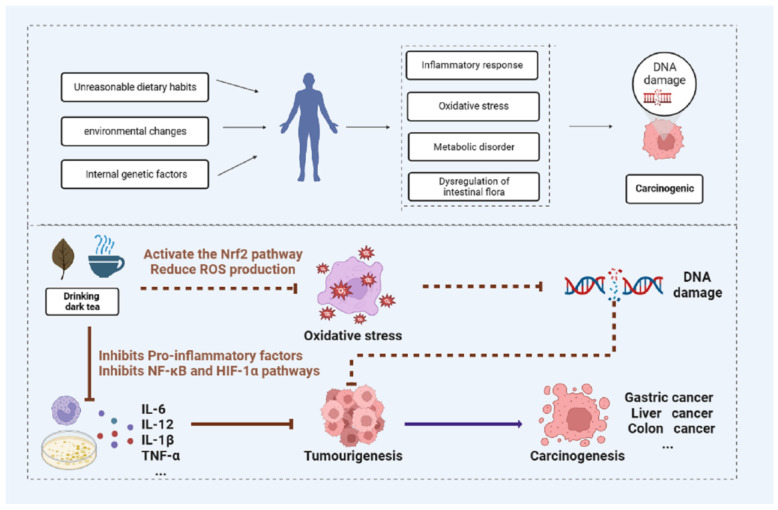
Possible anti-cancer mechanisms of dark tea.

As shown above, the anti-cancer effects of dark tea have been extensively studied, and their possible mechanisms are shown in Figure 1 and Table 1 below.

### 4.1. Anti-Inflammation

Research shows that the development of cancer is closely related to the inflammatory response, and many cancers are caused by chronic inflammation [36,37]. Nuclear factor-kappa B (NF-κB) is a transcription factor involved in a variety of biological processes, including immunity and inflammation [38,39], and it can facilitate the aggressive phenotype and transcription of oncogenes in cancer cells. In a prospective cohort study, a long-term anti-inflammatory diet was found to improve survival for breast cancer survivors [40]. Dark tea has been shown to have an anti-inflammatory effect [41], and the prevention and treatment of cancer from the perspective of anti-inflammatory has been evaluated in many clinical studies and reports [42]. In the analysis of the therapeutic effect of six kinds of tea on the liver injury induced by carbon tetrachloromethane (CCl4) in mice, dark tea can inhibit the NF-κB pathway to be anti-inflammatory and reduce liver injury, while green tea, yellow tea, oolong tea, and white tea have no such effect [43]. TB extracted from Pu’er tea regulates the immunity of RAW264.7 macrophages and inhibits inflammation by inhibiting the NF-κB/MAPK/PI3K-AKT signaling pathway [44]. Dark tea has also been shown to alleviate colitis induced by dextran sodium sulfate and reduce the expression level of inflammatory factors, mainly by regulating the NF-κB and HIF-1α signaling pathways, regulating gut bacteria, and enhancing the synthesis of short-chain fatty acids such as butyrate [45]. In DSS model mice, the intervention of Pu’er tea extract (100 mg/kg) and the positive control drug 5-aminosalicylic acid (100 mg/kg) were both found to reduce the levels of TNF-α, IL-6, and other pro-inflammatory cytokines in the serum and colon of colitis mice, inhibit the activation of the NF-κB pathway, and down-regulate the expression of HIF-1α [46,47]. Pu’er significantly down-regulated the levels of inflammatory pathway proteins (MyD88, TLR4, p38MAPK, and *p*-NF-κB p65); therefore, drinking aged Pu’er tea (10 mg/kg BW per day for mice and 7 g/kg BW per day for humans) has a practical effect on alleviating and preventing the development of intestinal inflammation [48]. Fuzhuan brick tea extract (30 mg/kg and 60 mg/kg) significantly reduced DSS-induced rectal bleeding, shortened colon length, and reduced the production of inflammatory cytokines in mice [49]. Fuzhuan tea polysaccharide reduced the level of inflammatory cytokines IL-1β, IL-6, IFN-γ, and TNF-α in DSS-induced mice [50]. The anti-inflammatory effect of Fuzhuan brick tea has been shown by Dai et al. to alleviate diarrhea in mice, significantly reducing the level of the pro-inflammatory factor 5-hydroxytryptamine (5-HT) and increasing the expression of sodium hydrogen exchanger 3 (NHE-3). Compared with berberine (60 mg/kg) in the positive control group, the effects of Fuzhuan brick tea (2530, 1260 mg/kg) on diarrhea were more notable [51]. Research shows that colitis can lead to colon cancer. Hu et al. found that Pu’er tea can not only diminish the development of inflammation and reduce the level of pro-inflammatory factors but also promote the synthesis of short-chain fatty acid butyrate (BA). Additionally, BA protects the intestinal barrier, which can significantly reduce inflammatory factors (IL-1β and IL-6) and increase anti-inflammatory cytokine release (IL-10 and IL-22). In addition, BA helps reduce the up-regulation of PI3K/AKT/NF-κB pathway proteins by DSS [52]. Overall, these findings indicate that dark tea may have potential as a natural approach to preventing colon cancer by reducing inflammation.

**Figure 2:**
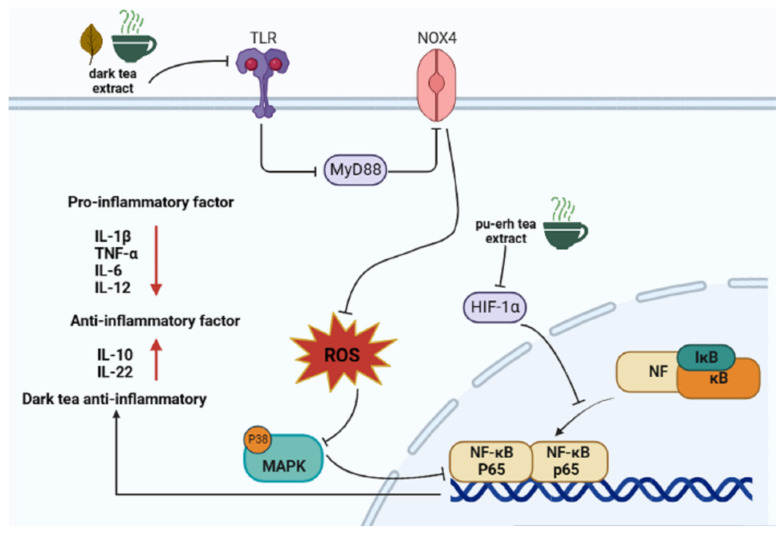
The anti-inflammatory pathway of dark tea.

In the model of inflammation induced by fat accumulation, dark tea has been shown in many studies to reduce the levels of pro-inflammatory factors and relieve the inflammatory response in the body. Liu et al. observed that Pu’er tea decreased the expression of the pro-inflammatory factor IL-6 in mice fed a high-fat diet (HFD) [53]. Xiao et al. found that ApoE knockout mice treated with Pu’er tea for 16 weeks had decreased relative mRNA expression levels of the pro-inflammatory factors IL-6, IL-12, and TNF-α in aortic tissues, and the activity of NF-κB was significantly reduced. This shows that Pu’er tea can inhibit lipid deposition in the blood vessel wall, reduce the inflammatory response, and finally inhibit plaque formation in the arterial wall [54]. Zhu et al. found that after Pu’er tea intervention, the levels of IL-6 and TNF-α were significantly reduced in both animal experiments and cell experiments, which reversed the inflammation caused by fat accumulation in the liver [55]. Similar conclusions were also verified by Cai et al., who showed that Pu’er tea significantly reduced the levels of TNF-α, Mcp1, and IL-1β in the liver of HFD-fed mice, which were regulated by IL-6/STAT3 signaling in the liver of mice [56]. Dark tea is also rich in tea polyphenols; it (TPs50%; 0.50 g/kg) can inhibit liver inflammation by inhibiting the expression levels of COX-2 and iNOS in an obese dog model. Increased COX-2 expression is seen in a variety of inflammatory diseases and human cancers, including lung and liver cancer [57]. Pu’er tea also significantly reduced the levels of IL-8, IL-1β, IL-6, and TNF in peripheral blood mononuclear cells (PBMC) obtained from healthy people’s blood samples (male = 6 and female = 6) [58]. The above content shows that dark tea can help reduce inflammation, possible pathways are shown in Figure 2 and the reduction of inflammation may reduce the risk of cancer, but it is not limited to this. It is worth noting that most research has focused on colitis and inflammation caused by fat accumulation. In view of this, future studies can focus on the anti-inflammatory effects of dark tea on other organ sites.

### 4.2. Antioxidation

Oxidative stress occurs when the production and removal of oxygen-free radicals in the body or cells are unbalanced. Oxidative stress is associated with the occurrence of a variety of diseases but also affects the evolution of cancer [59], stimulates tumorigenesis, and promotes the transformation and proliferation of cancer cells. Studies have demonstrated the antioxidant properties of dark tea [60,61], and antioxidants are negatively correlated with the occurrence of cancer [62]. Therefore, the consumption of dark tea may help prevent cancer by reducing oxidative stress in the body. Numerous researchers have used in vitro experiments to verify the antioxidant properties of dark tea. By using ABTS, FRAP, DPPH, HSA, SSA, and other experiments, the antioxidant properties of dark tea were confirmed. Zhao et al. found that dark tea had a strong antioxidant capacity; the total phenol content of dark tea was (81.43 ± 40.92 mg GAE/g DW), the FRAP value was (1472.27 ± 691.91 µmol Fe^2+^/g DW), and the TEAC value was (715.99 ± 352.02 µmol Trolox/g DW) [63]. Among them, polyphenols, including tea polyphenols, five catechins (EC, GC, GCG, CG, and EGCG), and two flavonoids (rutin and kaolin), showed a highly significant positive correlation with in vitro antioxidant activities [64]. Compared to mature Pu’er, Roda et al. found that the average antioxidant activity of raw Pu’er was higher, and the main contributors were polyphenols and flavonoids [65,66]. Zhang et al. found that phenolic components extracted from Pu’er have stronger antioxidant activity than vitamin C, and Pu’er may be an ideal natural antioxidant [67]. Guo et al. planned to compare the antioxidant capacity of tea polysaccharides extracted from different tea varieties and found that the total phenol and antioxidant activity of Pu’er tea polysaccharide (TP-4) were the highest through an in vitro antioxidant assay [68]. Zheng et al. extracted tea polyphenols and tea polysaccharides from Ya’an Tibetan tea, which were found to have antioxidant activity in vitro using DPPH [69]. In addition to the significant contribution of polyphenols to anti-oxidation, Su et al. isolated thealenol A from Pu’er tea and determined the antioxidant activity of teadenol A by DPPH and T-AOC in vitro [70]. The polysaccharides extracted from Fu Brick tea and Qingzhuan brick tea also have strong antioxidant activity in vitro [71,72]. Overall, these studies show significant antioxidant activity in dark tea, which is due to the presence of various active components such as polyphenols, flavonoids, and tea polysaccharides.

Several studies have investigated the antioxidant activity of dark tea in vitro. Hou et al. found that Pu’er tea extract diminished the level of MDA in kainic acid-treated PC12 cells, and inhibited the production of cellular ROS and lipid peroxidation [73]. Wang et al. demonstrated that Pu’er tea can remove ROS produced by human cancer cells (Caco2 and HepG2 cell lines) [74]. The 2S,3R-6-methoxycarbonylgallocatechin isolated from Anhua dark tea protected NRF2/ARE HEK293 cells from ROS via NRF2 activation [75]. The 8-C N-ethyl-2-pyrrolidinone-substituted flavan-3-ols extracted from Pu’er tea also possessed significant antioxidant activity and could prevent HMEC damage caused by H_2_O_2_ [76].

In vivo data also confirmed the antioxidant effect of dark tea. Dark tea extract reduced ROS levels in hematopoietic cells by inhibiting the expression of NOX4 and significantly improved the survival of mice exposed to 7.0 and 7.5 Gy total body irradiation [77]. Pu’er tea extract can improve the activities of antioxidant enzymes such as glutathione peroxidase (GSH-Px) and superoxide dismutase (SOD) in HFD rats and decrease the level of malondialdehyde (MDA), a lipid peroxidation product, in obese rats [78]. It can also inhibit oxidative stress and lipid peroxidation [79]. Braud et al. found that the Pu’er tea extract mixture decreased the production of the ROS marker O_2_^−^ in rat hepatocytes and also prevented t-BHP-induced mitochondrial oxidative stress [80]. Pu’er tea extract reduces serum MDA levels induced by quinocetone and increases the activities of GSH, SOD, and GPx. The main mechanism is to improve ERK phosphorylation levels and thus increase Nrf2/HO-1 pathway expression [81]. Similar results have also been confirmed. Wang et al. found that in QCT-induced mice, under the action of Pu’er tea extract, ROS accumulation is reduced, antioxidant activity is enhanced, and levels of antioxidant enzymes (SOD, GPx, and CAT) as well as non-enzymatic antioxidant GSH are also increased [82]. Zheng et al. found that Pu’er tea powder could reduce MDA and GSH levels and increase SOD and GSH-PX levels in rats induced by cisplatin [83]. Contrary to the findings of in vitro investigations, Cao et al. observed that dark tea had greater antioxidant capacity than green tea in vivo; dark tea extract reduced the level of malondialdehyde and increased the levels of superoxide dismutase, glutathione peroxidase, and glutathione in the liver of mice, indicating that tea has antioxidant and hepatoprotective activities [84].

There are many studies on the antioxidant properties of dark tea in vitro, and more in-depth cell and animal experiments should be carried out to further verify its antioxidant properties. Dark tea antioxidant summary are shown in Table 2. In animal experiments, there are many studies on high-fat diet modeling, and more disease models can be designed in the future to verify antioxidant activity. Moreover, most of the antioxidant studies only focus on the apparent enzymes without further exploring the pathways and mechanisms of antioxidants.

### 4.3. Inhibiting the Proliferation of Cancer Cells

Dark tea inhibits proliferation by inhibiting the cell cycle. In the study of breast cancer, proline dehydrogenase (PRODH) induced epithelial–mesenchymal transformation of cancer cells and increased cell proliferation. Xie et al. also focused on breast cancer and found that the proliferation of the human breast cancer cell line MDA-MB-231 was inhibited by Pu’er tea and that its mechanism was through activation of the JNK pathway [85].

Tumor cell growth is inhibited by down-regulating the S phase and causing stagnation in the G1 and G2 phases [86]. The inhibition of cell proliferation by Pu’er tea has been involved in numerous cancer studies. Pu’er tea induces G1 phase block in HepG2 cells, possibly through the activation of AMPK to improve the expression level of P21 [87]. EGCG inhibits the proliferation of oral cancer HSC-3 cells, causing them to stall in the G1 phase of the cell cycle [88]. In the gastric cancer cell line SGC-7901, Pu’er tea blocked the S phase of the cell cycle and significantly reduced the G2/M phase [89]. The new epigallocatechin gallate derivatives isolated from Anhua dark tea showed better antitumor activity than EGCG and inhibited the proliferation of lung cancer cells HCC827-Gef [29]. In human melanoma cells A375, TB in Pu’er tea inhibited their proliferation, causing DNA loss and inducing apoptosis; meanwhile, in a zebrafish xenotransplantation model of A375 cells, TB inhibited tumor growth, and the effect of TB was cancelled only by simultaneously eliminating p53 and p65. It is confirmed that it is mediated by the p53/NF-κB signaling pathway [31]. TB (500 μg/mL) can also significantly inhibit the proliferation of colon cancer cells (HT-29) through long-term oxidative stress [90]. Chen et al. conducted a population study and found that long-term Pu’er tea consumption down-regulates α9-nicotine-acetylcholine receptor (α9-nAchR) expression and protects against the formation of foam cells induced by nicotine inhalation due to smoking, which is beneficial to reduce the risk of lung cancer [91].

### 4.4. Promoting Cancer Cell Apoptosis

Microbial fermentation improves the bioavailability of dark tea components. It was found that methylxanthine isolated from Pu’er tea contributed to the anti-tumor activity of Pu’er tea and showed concentration-dependent growth inhibition on two cancer cell lines, MDA-MB-231 and HT-29 [92]. Zhao et al. found that fermented Pu’er tea increased the number of HT-29 colon cancer cells and human umbilical vein endothelial cell apoptotic bodies by DAPI staining and flow cytometry analysis. The expression of Bax, caspase-9, and caspase-3 mRNA increased, and the expression of Bcl-2 decreased [93]. Yao et al. used a weak tea polyphenol base to prepare theabrownins and found that it could induce apoptosis in HT-29 cells. They also explored the possible mechanism of inducing REDOX imbalance in cancer cells [94]. In addition, Liang et al. conducted in vivo experiments to explore the anti-cancer effect of theabrownins. TB promoted apoptosis of cancer cells, and the pathway was found to reduce phosphorylation of phosphatidylinositol 3-kinase (PI3K) and protein kinase B (Akt) [95]. Xiao et al. studied the effect of TB on lung cancer and found that TB induced apoptosis of lung cancer cell H1299 and inhibited tumor growth in zebrafish, which was a p53-independent mechanism mediated by the activation of the MAPK/JNK signaling pathway [96]. Zhao et al. found that Pu’er tea can inhibit the proliferation of human tongue cancer TCA8113 and induce its apoptosis through the decrease in matrix metalloproteinase (MMPs) and the increase in tissue inhibitor of metalloproteinase (TIMPs) mRNA transcription. It was also verified in a mouse experiment that the tumor volume of mice treated with Pu’er tea was significantly reduced, and apoptosis was induced by up-regulating Bax and down-regulating Bcl-2 [34]. Xu et al. concentrated their views on the prevention and treatment effects of TB on liver cancer and found that TB induced apoptosis of liver cancer cells (Huh7), up-regulated the expressions of ASK1, *p*-JNK, *p*-c-Jun, and Bax, and down-regulated Bcl-2. The JNK signaling pathway was also verified in in vivo experiments with zebrafish [32]. Zhang et al. showed that using Pu’er tea in combination with cancer immunotherapy is an effective way to fight cancer [97].

**Figure 3:**
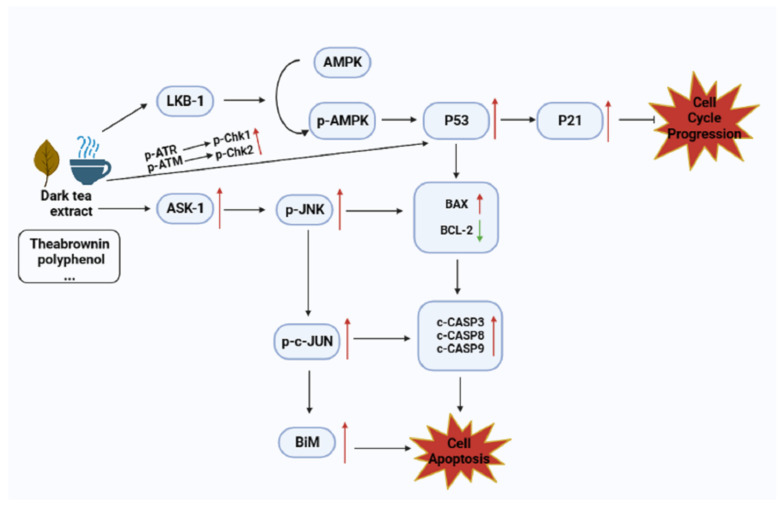
Dark tea acts on cancer cells. (Meaning: ↑: increase or ↓: decrease).

In conclusion, dark tea can prevent and treat cancer by inhibiting the proliferation, metastasis, and apoptosis of cancer cells. Possible pathways are shown in Figure 3. However, most of the studies only stay at the cellular and animal levels, and more population studies should be carried out.

### 4.5. Anti-Obesity

Obesity has become a widespread problem [98]. Various variables contribute to its occurrence, such as diet, metabolic abnormalities, and genetics [99]. In recent years, the number of obese people has been on the rise, and obesity is also closely related to the occurrence of certain cancers [100,101]. In a retrospective cohort study of 22,198 patients who underwent bariatric surgery between 2005 and 2012, patients who underwent bariatric surgery had an overall reduction in cancer incidence over 3.5 years of follow-up compared with matched participants who did not undergo surgery (HR = 0.67, 95% C.I. = 0.60–0.74, *p* < 0.001) [102]. Studies have indicated that excessive obesity can increase the risk of colon cancer and breast cancer, and obesity can promote tumorigenesis by stimulating inflammatory responses in the body [103]. Dark tea has been reported to lower lipids, which provides another perspective for the prevention and treatment of cancer [104,105].

Fu Brick Tea (200 mg/kg BW for 4 weeks) can reduce the levels of TG and TC in the serum of mice and alleviate the occurrence of alcohol-induced hepatic steatosis [106]. In the study of Pu’er tea, it was found to significantly reduce serum TG, TC, and LDL-C levels and increase NO content in mice fed a high-fat diet [107]. Similar conclusions were also verified in the zebrafish model, where different structures of TB from Pu’er tea were found to have lipid-lowering effects on high-fat-induced zebrafish. TB (1000 μg/mL) was able to reduce lipid levels in high-fat zebrafish to 51.57%, better than positive control simvastatin (0.06 μM) [108]. Ma et al. found that Pu’er tea can combine with bile salts to expel cholesterol from the body and stimulate the continuous conversion of cholesterol into bile salts to achieve a lipid-lowering effect [109]. Most studies on the lipid reduction of Pu’er tea have been conducted from the perspective of the regulation of obesity-related gene expression, such as farnesol X receptor (FXR), which is involved in the control of bile acid (BA) synthesis and hepato-intestinal circulation, and the activation of liver FXR and the inhibition of intestinal FXR are beneficial to obesity-related metabolic diseases [110]. Liupao tea (200 mg/kg BW for 8 weeks) can also reduce the levels of TG, TC, and LDL-C in the serum of obese mice and increase the level of HDL-C. The expression levels of PPAR-α, LPL, CPT1, and CYP7A1 were up-regulated, while the protein expression levels of PPAR-γ were down-regulated [111]. Xu et al. found that TB and tea polysaccharides inhibited lipase [112]. TB extracted from Fuzhuan tea can reduce the weight and white adipose tissue weight of HFD mice [113]. Fuzhuan tea and Ya’an Tibetan tea can improve obesity, prevent dyslipidemia, and reduce weight gain [114,115]. Lv et al. used different substances to extract the active components from Pu’er tea and found that flavonoids in Pu’er tea contributed the most to lipid lowering through high-throughput screening of cell models PPARδ, PPARγ, FXR, and LXR [116]. The view that polyphenols in Pu’er tea inhibit cholesterol synthesis in HepG2 cells was confirmed by Lu et al. [117]. Yue et al. found that Pu’er tea could inhibit the activity of pancreatic lipase in high-fat mice, down-regulating SREBP-1c and FAS and enhancing LDLR and CPT-1α to promote fat consumption [118]. Similarly, Ye et al. found that Pu’er tea also down-regulates the expression of LXRα, FAS, SREBP-1c, and PPARγ [119].

In addition to the mouse model, rat, rabbit, and Caenorhabditis elegans models were also used. Liang et al. found through rats and male rabbits that Pu’er tea can mediate lipid metabolism by inhibiting key enzyme activities, and Pu’er tea down-regulates Lp-PLA2, HMGR, and PL and up-regulates LCAT activity [120]. Huang et al. found that Pu’er tea decreased FAS expression and increased AMPK phosphorylation to improve fructose-induced hyperlipidemia [121]. LO2 cells and Caenorhabditis elegans were studied by Su et al., and it was found that Pu’er tea could relieve the expression of lipid accumulation and metabolism transcription factors such as PPARα, CD36, Plin2, Scd1, activate the SIRT1-FOXO pathway to inhibit the expression of SREBP-1c and FAS, and inhibit systemic lipidation in Caenorhabditis elegans [122]. Cao et al. focused their research on preadipocytes and found that Pu’er tea inhibited the proliferation and differentiation of 3T3-L1 preadipocytes because the transcription factors peroxisome proliferator-activated receptor-γ and CCAAT/enhancer binding protein-α were down-regulated during differentiation [123]. Moreover, the lipid-lowering effect of Pu’er tea is also closely related to intestinal flora, which was verified by Kuang et al. [124]. Pu’er tea was orally administered to obese mice for 9 weeks at a dose of 1000 mg/kg BW (equivalent to drinking 13–15 g tea per day for adults), and atorvastatin was administered at a dose of 10 mg/kg BW per day to inhibit obesity [53].

Dark tea has also been shown to inhibit obesity in human studies. Pu’er tea extract (3 g/day) taken daily for 20 weeks can significantly reduce body weight and improve blood lipid status in people with hyperlipidemia [125]. In healthy men aged 24–32 years, taking Pu’er tea powder (50 mg/kg) daily for 4 weeks can reduce hyperlipidemia [23]. In addition, Pu’er tea extract (Pu’er tea polyphenol 32.48 mg, eq. gallic acid) can also reduce blood sugar levels after meals [126]. In a multicenter, cross-sectional study, it was found that about 62% of Chinese people drink tea, mainly dark, black, and green tea. Daily tea consumption was inversely associated with the risk of diabetes in women, the elderly, and the obese. The prevalence of newly diagnosed diabetes in green, black, and dark tea drinkers was 9.8%, 5.8%, and 5%, respectively. Those who drank dark tea (Pu’er) had the lowest prevalence of newly diagnosed diabetes [127].

**Figure 4:**
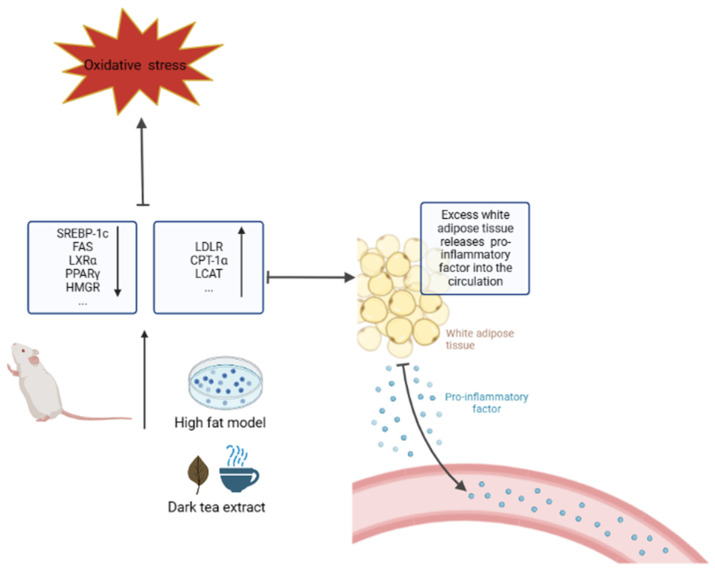
Dark tea reduces the production of pro-inflammatory factors by inhibiting obesity.

In general, dark tea has a lipid-lowering effect, which can reduce the production of pro-inflammatory factors in obese people, inhibit the growth and spread of tumors, and reduce the risk of cancer. Possible pathways are shown in Figure 4. It can also reduce the risk of cancer by reducing oxidative stress and DNA damage. The specific mechanism by which obesity reduces the incidence of cancer needs further study.

### 4.6. Regulating Intestinal Flora

It is shown that the imbalance of ecological disruption and the subsequent gut microbes in the intestine may lead to a variety of pathological changes and also lead to the occurrence of cancer [128,129]. While dark tea has been shown to modulate gut microbiota [130,131].

The polyphenols in dark tea can regulate the intestinal microbiota in animal and human experiments, it has been confirmed by many people that tea can increase the α and β diversity of the gut microbiota in animals [132]. Gao et al. found that active ingredients such as polyphenols and caffeine in Pu’er tea increased the abundance of *Akkermansia muciniphila* and *Faecalibacterium prausnitzii* in mice on a high-fat diet, thereby improving obesity and inflammation [133]. Additionally, TB plays an important role in regulating the intestinal flora. Deng et al. found that TB can promote the proliferation of beneficial microbiomes, such as *Lactobacillus* and *Lachnospiraceae*_*NK4A136*_group [134]. Yue et al. found that Pu’er tea increased the composition of *Actinobacteria* and *Proteobacteria* in rats with a high-fat diet, and the effect of TB on diabetic rats mainly depended on the targeted regulation of intestinal microorganisms [135]. Yue further induced rats with high sugar, high fat, and high salt and found that the prognosis of TB and the ratio of *Firmicutes* to *Bacteroidetes* (F/B) decreased significantly, which promoted the propagation of *Bacteroidetes* and inhibited *Firmicutes* [136]. Su et al. found that Pu’er tea can regulate the gut microbes in colitis mice and increase the abundance of beneficial bacteria, such as *Muribaculum*, *Lactobacillus*, *Rikenellaceae,* and *Lachnospiraceae*; the abundance of harmful bacteria, *Romboutsia* and *Turicibacter,* decreased, which provided a safe and effective new strategy for the prevention and treatment of colitis and reduced the probability of colon cancer [137]. In addition, Huang et al. found that, in addition to regulating the composition and proportion of intestinal microbes, SCFAs produced by intestinal microbiota also played an important role [138].

Whether there are individual differences in the regulation of intestinal flora and the specific mechanism of action between the active components of dark tea extract and intestinal flora needs to be further explored.

**Table 1:** Possible anti-cancer mechanisms of dark tea. (Meaning: ↑: increase or ↓: decrease).

Mechanisms	Related Genes/Proteins	Reference
Anti-inflammatory	TLR, HIF-α, NF-κB, MAPK ↓	[41,42,43,44,45,46,51]
Antioxidant	MDA, ROS ↓ SOD, GSH ↑	[78,81,82,83]
Inhibit proliferation	JNK, AMPK ↑	[84,85,86,87,88]
Promote apoptosis	BCL-2 ↓ Caspase-9, BAX, caspase-3 ↑	[92,93,94,95,96]
Inhibit obesity	SREBP, FAS, HMGR, LXRα ↓ LDLR, LCAT, CPT-1α ↑	[106,110,111,115,117,120]
Regulate intestinal flora	*Akkermansia muciniphila* ↑ *Faecalibacterium prausnitzii* ↑ *Lactobacillus*, *Actinobacteria* ↑ *Proteobacteria* ↑	[132,133,134,135,136]

**Table 2:** Antioxidation of dark tea and its pathways.

	Research Method/Model	Active Component	Conclusions
In vitro study	FRAP, TEAC	Dark tea extract	1472.27 ± 691.91 µmol Fe^2+^/g DW 715.99 ± 352.02 µmol Trolox/g DW 81.43 ± 40.92 mg GAE/g DW [63]
	FRAP, DPPH ABTS, HAS, SSA	Pu’er tea extract (PRT)	As the pertinent antioxidants in PRT, EC, GC, GCG, CG, EGCG, rutin, and kaempferol contributed to the antioxidant activities [64]
	DPPH, SARSA, ABTS, ORAC, FRAP	Ripe and raw Pu’er tea	Raw Pu’er tea extract has better antioxidant capacity [65]
	ABTS, FRAP	Pu’er tea extract	Tea polysaccharides, tea polyphenols, and proteins are thought to be accountable for the biological activity of Pu’er tea [66]
	DPPH, ABTS	Tea polysaccharides	Compared with 12 kinds of tea, the highest total phenolic and protein content as well as the best antioxidant were found in pu’er tea polysaccharide [68]
In vitro study	DPPH	Ya’an Tibetan tea	Tea polyphenols in Ya’an Tibetan tea showed higher antioxidant activity than tea polysaccharides [69]
	DPPH, T-AOC	Teadenol A	Thealenol A isolated from fermented Pu’er tea has antioxidant properties and is an important bioactive component [70]
	ABTS, SOA, DPPH	Fuzhuan dark tea polysaccharides (DTPS)	The content and molecular weight of uronic acid may be the important factors affecting the oxidation resistance of DTPS [71]
	ROS, MDA determination	Gamma-aminobutyric acid (GABA) in Pu’er tea	Pu’er tea extract (1, 10 μ g/mL) and GABA (0.1, 1, 10 μM) decreased ROS production and lipid peroxidation in PC12 cells in a dose-dependent manner [73]
	ROS determination	Theabrownins (TBs)	At the concentration range of 1.25 to 6.25 mg/mL, Pu’er tea has obvious intracellular ROS clearance ability on human cancer cells (Caco2, HEp2, Hep G2 cell lines), and theabrownins are the main contributor [74]
	ROS determination	2S,3R-6-methoxycarbonylgallocatechin (MCGE)	MCGE protects cells from the production of ROS in UVB-exposed keratinocytes (HACats) by activating the Nrf2 pathway [75]
	Cell viability	8-C N-ethyl-2-pyrrolidinone substituted flavan-3-ols	The 8-C N-ethyl-2-pyrrolidinone substituted flavan-3-ols possessed significant antioxidant activity and could prevent HMEC damage caused by H_2_O_2_ [76]
	Mice exposed to 7.0 and 7.5 Gy total body irradiation	Dark tea extract	Dark tea extract reduced ROS levels in hematopoietic cells by inhibiting the expression of NOX4 [77]
	Obese rat model	Pu’er tea	Pu’er tea increased the activity of antioxidant enzymes such as SOD and GSH-Px, while decreased the level of lipid peroxidation product MDA in obese rats [78,79]
	t-BHP oxidative stress-induced rat hepatocyte model	Pu’er tea	Pu’er tea extract decreased the production of ROS marker O_2_^−^ in rat hepatocytes and prevented t-BHP induced mitochondrial oxidative stress [80]
	SD rats Balb/c mice	Pu’er tea	Pu’er tea reduced quinocetone-induced oxidative stress [81,82]
	Wistar rats	Pu’er tea	Pu’er tea decreased MDA and GSH, and increased SOD and GSH-Px levels [83]
	Mice with acute alcoholic liver injury	Different dark tea extracts	Dark tea has greater in vivo antioxidant activity than green tea [84]

## 5. Expectation and Prospect

To sum up, the fact that dark tea can prevent and treat cancer has been proven by many studies, but there are still problems to be solved and improved. There are many studies on colitis and colon cancer in dark tea, and more research on the prevention and treatment of cancer in other parts of the body should be carried out in the future. At the same time, most of the studies focus on the apparent but fail to deeply explore the pathways of cancer prevention and treatment, and there is a lack of holistic connection between each anti-cancer pathway.

In a prospective study of 532,949 participants, higher levels of tea drinking were associated with a lower risk of bladder cancer (compared with no tea consumption: HR = 0.87, 95% C.I. = 0.77–0.98 for low consumption; HR = 0.86, 95% C.I. = 0.77–0.96 for moderate consumption; HR = 0.84, 95% C.I. = 0.75–0.95 for high consumption) [139]. In the European Prospective Investigation into Cancer and Nutrition, 201 cases of hepatocellular carcinoma were identified among 486,799 men/women followed for 11 years, and it was found that increased tea intake was associated with a lower risk of hepatocellular carcinoma [140]. Studies have found that drinking three cups of fermented dark tea a day can reduce the risk of coronary heart disease and diabetes [141]. And there have been many prospective studies on the anti-cancer effects of black tea and green tea. Increasing the intake of green tea and black tea can reduce the risk of lung cancer, gynecological cancer, bladder cancer, etc., and its anti-cancer effect is mainly attributed to tea polyphenols, such as epigallocatechin-3-gallate and theaflavins [142,143,144,145]. However, there are few prospective studies focusing on the anti-cancer effect of dark tea, which can be increased in the future to make dark tea more convincing in preventing and managing cancer.

## 6. Conclusions

Cancer has become the primary problem threatening human health, and the number of cancer patients is also increasing rapidly [146]. Therefore, the prevention and treatment of cancer have become two of the most promising directions. At present, the main treatment for cancer is radiotherapy and chemotherapy [147,148], but these methods are harmful to the human body [149], and it is more realistic and reliable to prevent cancer through daily dietary intake.

This paper reviewed the effect of dark tea on cancer prevention and treatment and established the interaction and corresponding mechanism. The summary shows that the main mechanisms of dark tea preventing cancer are anti-inflammatory, antioxidant, inhibiting cancer cell proliferation, inducing cancer cell apoptosis, anti-obesity, and regulating intestinal flora. Daily consumption of dark tea can achieve cancer prevention by inhibiting pro-inflammatory cytokines TNF-α, IL-1β, and IL-6 and increasing anti-inflammatory cytokines IL-10 and IL-22, which is mainly attributed to the down-regulation of the NF-κB signaling pathway. The treatment of dark tea can also effectively prevent cancer by increasing the activity of the antioxidant enzymes SOD, CAT, and GSH-Px, removing ROS produced by cells, and decreasing the level of MDA. The antioxidant pathway is mainly attributed to increasing the level of ERK phosphorylation and increasing the expression of the Nrf2/HO-1 signaling pathway. Dark tea’s inhibition of cancer cell proliferation and induction of apoptosis have also been verified by many studies, which are mainly mediated by the MAPK/JNK signaling pathway. Dark tea also reduces the levels of serum TC, TG, and LDL-C, mainly inhibits the synthesis of fatty acids and cholesterol in the body, and promotes the realization of excretion, thus reducing obesity-related metabolic diseases and preventing cancer. At the same time, dark tea can also regulate intestinal flora, improve the diversity of intestinal microbes α and β, promote the reproduction of bacteroides, and inhibit firmicutes to effectively prevent cancer.

Theabrownin, tea polyphenol, and tea polysaccharide are the main active substances in dark tea. Although a large number of in vitro and in vivo studies have confirmed the positive effect of dark tea extract on human health, complete research on the exact molecular mechanisms of various active ingredients related to their corresponding anti-cancer abilities is still lacking. In addition, as is known to all, the bioavailability of dark tea in the human body is poor [150,151], and there are few studies on the anti-cancer effects of specific active substances in dark tea. Whether the anti-cancer effect of dark tea is more effective with a single component or the synergistic effect of different components needs to be further confirmed. It is important to note that dark tea, a natural food, can be highly recommended as a nutritional supplement for cancer prevention and management with few side effects.

## Data Availability

No new data were created.

## Abbreviations

TB, theabrownin; PI3K, phosphatidylinositol-3 kinase; Akt, protein kinase B; HDL-C, high-density lipoprotein cholesterol; TG, triglyceride; LDL-C, low-density lipoprotein cholesterol; TC, total cholesterol; GSH, glutathione; SOD, superoxide dismutase; MDA, malondialdehyde; ROS, reactive oxygen species; HMGCR, 3-hydroxy-3-methylglutaryl-coenzyme A reductase; FASN, fatty acid synthase; PCSK9, proprotein convertase subtilisin/kexin type 9; ACC, acetyl-CoA carboxylase; SREBP, sterol regulatory element-binding protein; LDLR, low-density lipoprotein receptor; NF-κB, nuclear factor-kappa B; DSS, dextran sodium sulfate; TNF-α, tumor necrosis factor-α; IL-6, interleukin-6; HIF-1α, hypoxia-inducible factor-1α; IL-1β, interleukin-1β; IL-10, interleukin-10; IL-22, interleukin-22; EGCG, (−)-epigallocatechin gallate; EC, (−)-epicatechin; EGC, (−)-epigallocatechin; CG, (−)catechingallate; GC, (−)-gallocatechin; IFNγ, interferon-γ; TLR4, toll-like receptor 4; COX-2, cyclooxygenase-2; MyD88, myeloid differentiation factor88; MAPK, mitogen-activated protein kinases; TEAC, Trolox equivalent antioxidant capacity; STAT3, signal transducer and activator of transcription 3; INOX, inducible nitric oxide synthase; FRAP, ferric-reducing antioxidant power; DPPH, 1,1-diphenyl-2-picrylhydrazyl(DPPH)free-radical scavenging ability; ABTS, 2,2′-azino-bis (3-ethylbenzothiazoline-6-sulfonic acid) diammonium salt (ABTS)•+ scavenging ability; HAS, hydroxyl radical scavenging ability; SSA, superoxide anion radical scavenging ability; TAOC, totalantioxidantcapacity; PRODH, prolinedehydrogenase; DAPI,4′,6-diamidino-2′-phenylindole; BAX, BCL2-associated X; BCL-2, B-cell lymphoma-2; PPARγ, peroxisome proliferator-activated receptor γ; LXRα, liver X receptor α; Lp-PLA2, lipoprotein-associated phospholipase 2; LCAT, lecithin-cholesterolacyltransferase; CD36, platelet glycoprotein 4; Plin2, perilipin2; Scd1, stearyl coenzyme A desaturated enzyme.
